# Do the benefits of IDH mutations in high-grade glioma persist beyond the first recurrence? A multi-institutional retrospective analysis

**DOI:** 10.1007/s11060-025-05049-2

**Published:** 2025-04-22

**Authors:** Anne R. Lally, Sayak R. Ghosh, Isabella L. Pecorari, Joshua Reynolds, Alexander Ledet, Sabrina Begley, Elizabeth Juarez Diaz, Eric Zhu, Karan Joseph, Kyle McGeehan, Michael Schulder, Tanner Johanns, Yonah C. Ziemba, Vijay Agarwal

**Affiliations:** 1 Department of Neurological Surgery, Montefiore Medical Center, The University Hospital for the Albert Einstein College of Medicine, 3316 Rochambeau Avenue, Bronx, NY USA; 2 Department of Neurosurgery, Donald and Barbara Zucker School of Medicine at Hofstra Northwell, Hempstead, 500 Hofstra Blvd, NY 11549 USA; 3 Department of Oncology, Washington University at St. Louis, St. Louis, 660 S Euclid Ave, MO 63110 USA; 4 Department of Pathology and Laboratory Medicine, Donald and Barbara Zucker School of Medicine at Hofstra Northwell, Hempstead, 500 Hofstra Blvd, NY 11549 USA

**Keywords:** Glioblastoma, IDH, Recurrence, Progression, Multi-institutional

## Abstract

**Purpose:**

Recurrence is inevitable in both IDH wild-type glioblastoma and IDH-mutant WHO grade 3 or 4 astrocytoma. While IDH-mutant astrocytomas are associated with longer survival and delayed first progression, less is known about disease course beyond initial treatment. This study examines whether IDH mutation status influences time to second recurrence and identifies additional predictors of recurrence intervals.

**Methods:**

This retrospective, multi-institutional study included adults diagnosed with pathologically confirmed high-grade glioma (HGG) between 2015 and 2020. HGG refers to IDH-mutant WHO grade 3 or 4 astrocytomas and IDH wild-type glioblastomas, consistent with WHO CNS5 criteria. Demographics, treatment, extent of resection, and molecular markers were analyzed. Time-to-recurrence was calculated per RANO 2.0 criteria. Statistical tests included Mann‒Whitney U, Fisher’s exact, and Cox regression.

**Results:**

Among 319 patients, 121 met inclusion criteria. Fourteen (11.6%) had IDH-mutant astrocytomas, and 107 (88.4%) had IDH wild-type glioblastomas. Mean time to first recurrence was significantly longer in IDH-mutant patients (17.5 months) than IDH wild-type (9.8 months, p = 0.0130). Mean time-to-second recurrence was not significantly different (IDH-mutant: 10.8 months, IDH wild-type: 8.1 months, p = 0.176). Multivariate analysis found IDH wild-type status (p = 0.0491) and Black race (p = 0.0238) predicted shorter time to first recurrence.

**Conclusions:**

IDH mutation status significantly affects time to first but not second recurrence. This study offers insight into recurrence patterns and highlights disparities in disease progression.

## Introduction

High-grade gliomas (HGGs) are the most common primary malignant brain tumors in adults [1–4]. In this study, HGG refers to IDH-mutant WHO grade 3 or 4 astrocytomas and IDH wild-type glioblastomas, consistent with the 2021 WHO CNS5 classification [3]. This update separates IDH-mutant astrocytomas from IDH wild-type glioblastomas due to their distinct prognoses [3, 5]. IDH mutations define a subgroup of diffuse gliomas—including WHO grade 3 and 4 astrocytomas—that present at younger ages and confer improved survival relative to IDH wild-type glioblastomas [5–10].

Despite this survival advantage, recurrence is nearly universal. Standard treatment includes maximal safe resection, radiotherapy, and temozolomide [11, 12], yet strategies at recurrence vary. Earlier studies reported progression-free survival (PFS) between 5.6 and 11.2 months in glioblastoma [13, 14], though these figures largely predate molecular classification. More recent studies show significantly longer PFS in IDH-mutant astrocytomas compared to IDH-wildtype glioblastomas [15, 16]. Recurrence timing may reflect tumor biology—shorter intervals suggest aggressiveness, while longer ones may indicate responsiveness to therapy. However, the prognostic significance of recurrence timing beyond the first progression remains unclear.

Though IDH-mutant astrocytomas generally offer longer survival, it is unknown whether their recurrence patterns differ meaningfully from IDH wild-type glioblastomas. Differences in metabolism, immune microenvironments, and treatment sensitivity may play a role [17, 18], but studies evaluating recurrence intervals beyond first progression remain limited due to the rarity of IDH-mutant cases. This study examines whether IDH mutation status influences recurrence timing beyond initial progression and explores additional predictors of recurrence intervals. We hypothesize that although IDH-mutant astrocytomas show longer initial PFS, second recurrence intervals will converge due to acquired resistance and cumulative mutations. These insights may inform personalized surveillance and salvage strategies in recurrent HGG.

## Methods

This IRB-approved retrospective study analyzed deidentified data from patients treated at Montefiore Medical Center, Northwell Health, and Barnes-Jewish Hospital. As part of a multi-institutional collaboration, we identified adults (≥ 18 years) diagnosed with WHO grade 3 or 4 gliomas between 2015 and 2020—a period selected to reflect current classification and treatment protocols. Consistent with the 2021 WHO CNS5 criteria, HGGs in this study included IDH-mutant grade 3 or 4 astrocytomas and IDH wild-type glioblastomas; IDH-mutant oligodendrogliomas were excluded.

Of 319 patients, 121 met inclusion criteria: documented IDH status and adequate follow-up through first recurrence. Recorded variables included age, sex, race, ethnicity, MGMT methylation, treatment, and extent of resection. Due to high missingness—particularly in the IDH-mutant subgroup—MGMT methylation was excluded from primary multivariate models and analyzed separately in complete-case analyses.

Surgical intervention was categorized as gross total resection (GTR), subtotal resection (STR), or biopsy. GTR was defined as ≥ 98% resection of contrast-enhancing tumor per postoperative MRI (typically within 72 h), in line with prior literature [19]. STR was defined as visible residual enhancement; biopsy referred to diagnostic sampling without debulking. Chemotherapy and radiation were recorded as binary variables. While protocols varied across institutions, all care was NCCN-concordant. Cases with biopsy-only diagnosis or indeterminate MGMT were retained if clinically appropriate per NCCN guidelines.

Recurrence intervals were calculated using imaging dates. Recurrence was defined using Response Assessment in Neuro-Oncology (RANO) 2.0 criteria and assessed by radiologists at each institution based on radiology reports incorporating volumetrics, contrast enhancement, and clinical judgment [20]. No re-measurements were performed by the study team.

### Statistical analysis

Data were analyzed using GraphPad Prism 10 and Microsoft Excel (Office 365). Mann–Whitney U tests were used for univariate comparisons between IDH-mutant astrocytoma and IDHwt GBM groups. Fisher’s exact tests assessed associations between IDH status and categorical clinical variables. Statistical significance was defined as p < 0.05. Cox proportional hazards models were used for multivariate analysis. Only variables that were significantly different between groups in univariate analysis were included to reduce overfitting. MGMT methylation was excluded from primary models due to missingness and assessed separately. To evaluate institutional treatment variability, an exploratory model incorporating re-resection and chemotherapy at recurrence was performed but not retained due to lack of significance.

## Results

### Patient cohort demographics

Of 319 patients treated for HGG, 121 met inclusion criteria (Table 1). Fourteen (11.6%) had IDH1-mutant WHO grade 3 or 4 astrocytomas, and 107 (88.4%) had IDH1 wild-type glioblastomas (IDHwt GBM). Among IDH-mutant cases, 3 were grade 3 and 11 grade 4. The mean age at diagnosis for IDH1-mutant astrocytomas was 46.1 years (95% CI: 39.3–52.9), with a median of 48.0 [IQR: 34.3–56.3]. For IDHwt GBM, the mean was 62.5 years (95% CI: 61–65), and the median was 63.0 [IQR: 55.0–71.0] (p < 0.0001).

**Table 1:** Patient Demographics and Treatment Summary

		Whole Cohort	IDHmut Astrocytoma	IDHwt GBM	P-value
		Mean	Mean	Mean	
Sample Size		121	14	107	
Sex (% Female)		43.00%	50.00%	42.10%	0.7751
Age at Diagnosis		60.6 (13.2)	46.1 (11.8)	62.5 (12.1)	< 0.0001*
Race (%)	White	52.90%	21.40%	57.00%	0.0202*
	Black	12.40%	14.30%	11.20%	0.665
	Asian	6.60%	7.10%	6.50%	> 0.9999
	Other	21.50%	42.90%	18.70%	0.0756
	Unavailable	7.40%	14.30%	6.50%	0.2787
WHO Grade (IDH-Mutant Astrocytoma Only)	Grade 3		21.43%		
	Grade 4		78.57%		
MGMT Status	Methylated (%)	21.50%	21.40%	21.50%	> 0.9999
	Unmethylated (%)	40.50%	28.60%	42.10%	0.3973
	Unavailable (%)	38.00%	50.00%	36.40%	0.3851
Treatment (%)	Chemotherapy	90.90%	100.00%	89.70%	0.3592
	Radiation	91.70%	92.90%	91.60%	> 0.9999
Extent of Surgical Resection (%)	Gross Total Resection (GTR)	38.80%	28.60%	40.20%	0.5623
	Subtotal Resection (STR)	30.60%	21.40%	31.80%	0.5471
	Biopsy	21.50%	35.70%	19.60%	0.1777
	Unavailable	9.10%	14.30%	8.40%	0.6147

Standard deviation (SD) is reported only for continuous variables and is in parentheses next to mean

Percentages are used for categorical variables

Statistical comparisons were made using Fisher’s exact or Mann–Whitney U tests where appropriate

^*^indicates statistical significance of p < 0.05

Fifty percent of IDH-mutant patients were female, and 21.4% were White, 14.3% Black, 7.1% Asian, and 42.6% other. For IDHwt GBM, 42.1% were female, 57.0% White, 11.2% Black, 6.5% Asian, and 18.7% other. The proportion of White patients differed significantly (p = 0.0202).

No significant differences were seen in tumor management. GTR was performed in 28.6% of IDH-mutant astrocytomas vs. 40.2% of IDHwt GBMs, STR in 21.4% vs. 31.8%, and biopsy alone in 35.7% vs. 19.6%.

### IDH1 mutation status and time to first recurrence

**Fig. 1:**
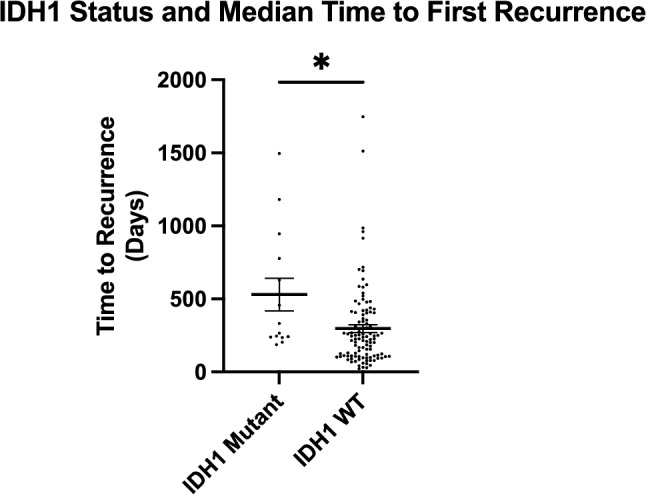
Patients with IDH1 Mutant HGG Have a Longer Median Time to First Recurrence than IDH1 Wild-Type. (*) indicates statistical significance of p < 0.05

The mean time to first recurrence was 531 days (17.5 months; 95% CI: 9.5–25.4) for IDH-mutant astrocytomas, with a median of 299 days (9.8 months; IQR: 238–820). For IDHwt GBM, the mean was 297 days (9.8 months; 95% CI: 8.0–11.5), median 228 days (7.5 months; IQR: 109–406). This difference was significant (p = 0.0130, Mann–Whitney U) (Fig. 1).

The right-skewed distribution in the IDH-mutant group, driven by a subset with > 1200 days PFS, elevated the mean above the median. Both measures are reported to reflect this variability.

In multivariate analysis (Table 2), IDH1 mutation remained a significant independent predictor of longer PFS (estimate: 183.0 days; 95% CI: –365.2 to –0.72; p = 0.0491). Black race was also associated with shorter PFS (estimate: –221.6 days; 95% CI: –413.2 to –30.1; p = 0.0238). Other variables were not significant. These race-based findings should be interpreted as exploratory.

**Table 2:** Potential predictors on time to first recurrence in high grade Glioma

Variable	Estimate	Standard error	95% CI (asymptotic)	P value
IDH1 mutation [Wild-Type]	− 183	92	− 365.2 to − 0.7151	*0.0491
Race [Black]	− 221.6	96.72	− 413.2 to − 30.05	*0.0238
Race [White]	− 34.43	71.99	− 177.0 to 108.2	0.6334
Race [Unavailable]	− 69.55	117.8	− 302.9 to 163.8	0.556
Race [Asian]	− 23	120.3	− 261.4 to 215.4	0.8488
Age at diagnosis	− 3.119	2.358	− 7.791 to 1.553	0.1887

^*^Indicates statistical significance to p < 0.05

### MGMT methylation and first recurrence

**Fig. 2:**
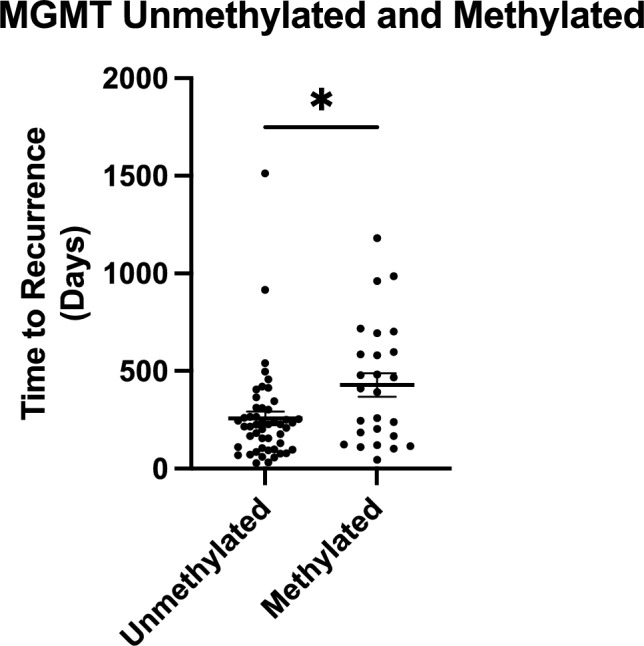
Patients with MGMT Methylated HGG Have a Longer Median Time to First Recurrence than MGMT Unmethylated. (*) Designates p < 0.05

Among 75 patients with MGMT data, 49 were unmethylated and 26 methylated. Unmethylated tumors had a mean PFS of 258 days (8.5 months; 95% CI: 6.2–10.7) and median 226 days (7.4 months; IQR: 3.3–10.1). Methylated tumors had a mean of 429 days (14.2 months; 95% CI: 10.0–18.2) and median of 402 days (13.2 months; IQR: 5.1–20.4) (p = 0.0115) (Fig. 2).

In an exploratory analysis of 68 IDHwt GBM patients with MGMT data, unmethylated cases (n = 45) had a mean PFS of 254 days (8.4 months; 95% CI: 5.9–10.8) and median 215 days (7.1 months; IQR: 3.2–10.1). Methylated tumors (n = 23) had a mean of 414 days (13.7 months; 95% CI: 9.6–17.6) and median 411 days (13.5 months; IQR: 4.1–19.6) (p = 0.0117).

### Patient characteristics at second recurrence

Of 121 patients, 43 had a second recurrence: 8 IDH-mutant astrocytomas (18.6%) and 35 IDHwt GBMs (81.4%). IDH-mutant patients had a mean age of 46.9 years (95% CI: 36.8–56.9), median 48.5 [IQR: 33.8–58.8]; IDHwt GBM had a mean of 60.4 (95% CI: 57.0–64.0), median 61.0 [IQR: 53.0–68.0] (p < 0.006). No significant differences in surgical or medical management were observed (Table 3).

**Table 3:** Demographics and treatment summary of patients with a second recurrence of high-grade glioma

		Whole Cohort	IDHmut Astrocytoma	IDH1wt GBM	P-value
		Mean	Mean	Mean	
Sample Size		43	8	35	
Sex (% Female)		32.6%	50.0%	28.6%	0.4038
Age at Diagnosis		57.88 (12.47)	46.9 (12)	60.4 (11.3)	0.006*
MGMT Status	Methylated (%)	18.6%	12.5%	20.0%	> 0.9999
	Unmethylated (%)	37.2%	25.0%	40.0%	0.6882
	Unavailable (%)	44.2%	62.5%	40.0%	0.4319
Race (%)	White	62.8%	37.5%	68.6%	0.125
	Black	14.0%	25.0%	11.4%	0.3078
	Asian	4.7%	12.5%	2.9%	0.3411
	Other	14.0%	25.0%	11.4%	0.3078
	Unavailable	4.7%	0.0%	5.7%	> 0.9999
Initial treatment (%)	Chemotherapy	95.3%	100.0%	94.3%	> 0.9999
	Radiation	93.0%	87.5%	94.3%	0.4697
Extent of first surgical resection (%)	Gross total resection (GTR)	44.2%	50.0%	42.9%	> 0.9999
	Subtotal resection (STR)	20.9%	0.0%	25.7%	0.171
	Biopsy	27.9%	37.5%	25.7%	0.6649
	Unavailable	7.0%	12.5%	5.7%	0.4697
Second treatment (%)	Chemotherapy	79.1%	87.5%	77.1%	> 0.9999
	Radiation	20.9%	25.0%	20.0%	> 0.9999
	Surgery	53.5%	62.5%	51.4%	0.7041
Extent of second surgical resection (%)	Gross total resection (GTR)	34.8%	20.0%	38.9%	0.6214
	Subtotal Resection (STR)	43.5%	80.0%	33.3%	0.1269
	Biopsy	4.3%	0.0%	5.6%	> 0.9999
	Unavailable	17.4%	0.0%	22.2%	0.5392

Standard deviation (SD) is reported only for continuous variables and is located in parentheses

Percentages are used for categorical variables

Statistical comparisons were made using Fisher’s exact or Mann–Whitney U tests where appropriate

^*^Indicates statistical significance to p < 0.05

Initial surgery included GTR in 4 IDH-mutant patients, biopsy in 3, and missing data for 1; IDHwt GBM patients underwent GTR (n = 14), STR (n = 12), biopsy (n = 7), or had missing data (n = 3). At recurrence, surgery was performed in 62.5% of IDH-mutant and 51.4% of IDHwt GBM cases. Among IDH-mutant cases, 1 had GTR, 4 STR, and 3 biopsy; among IDHwt GBM, 7 had GTR, 6 STR, and 5 biopsy. All patients received NCCN-concordant care.

### Time to second recurrence

The mean time to second recurrence was 10.8 months (95% CI: 6.8–14.9) in IDH-mutant astrocytomas and 8.1 months (95% CI: 6.2–10.0) in IDHwt GBM. Medians were 10.2 months [IQR: 6.5–15.8] and 7.4 months [IQR: 4.3–10.1], respectively (p = 0.176).

Multivariate regression showed no significant associations: IDH1 status (− 97.0 days; 95% CI: − 244.3 to 50.2; p = 0.19), age at diagnosis (0.97 days/year; 95% CI: − 3.67 to 5.62; p = 0.68). An exploratory model including re-resection and chemotherapy at recurrence also showed no significant associations.

### MGMT and second recurrence

Among 25 patients with MGMT data at second recurrence, 8 were methylated, 17 unmethylated. The methylated group had a mean time of 11.6 months (95% CI: 5.9–17.1), median 10.5 months [IQR: 5.3–16.7]; the unmethylated group had a mean of 7.2 months (95% CI: 4.5–9.9), median 6.4 months [IQR: 3.3–8.5] (p = 0.1363).

Within the IDHwt GBM subgroup (n = 22), MGMT-methylated tumors (n = 7) had a mean time to second recurrence of 11.1 months (95% CI: 4.4–17.6), median 8.4 months [IQR: 4.8–17.3]. Unmethylated tumors (n = 15) had a mean of 7.9 months (95% CI: 4.7–10.9), median 7.1 months [IQR: 3.8–9.1] (p = 0.3509). These analyses remain exploratory due to limited sample size.

## Discussion

Patients with IDH-mutant astrocytomas generally have a more favorable prognosis than those with IDHwt glioblastoma [7, 13, 18, 21]. This retrospective study examined progression-free survival (PFS) from initial surgery to first recurrence, and from first to second recurrence, to evaluate whether IDH1 mutations confer ongoing benefit. While IDH1 mutations significantly predicted longer time to first recurrence, they did not affect the interval between first and second recurrences. These findings suggest that the early advantage associated with IDH1 mutations may diminish over time, potentially due to accumulating genetic or epigenetic alterations during malignant progression. Prior genomic studies show that recurrent gliomas—especially those treated with temozolomide—acquire new mutations and undergo molecular shifts, including hypermutation and epigenetic loss [22, 23]. This underscores the dynamic nature of glioma evolution and the need to adjust treatment strategies after first recurrence. The right-skewed distribution in IDH-mutant astrocytoma recurrence intervals, driven by a subset of long-surviving patients, raised the mean relative to the median. This heterogeneity highlights the biological variability of IDH-mutant astrocytomas and supports reporting both median and mean values to more fully capture clinical outcomes.

All patients in this study had primary HGG, defined as IDH-mutant WHO grade 3 or 4 astrocytomas or IDH wild-type glioblastomas, per the 2021 WHO CNS5 classification. Prior to CNS5, studies estimated the frequency of IDH1 mutations in primary glioblastoma at 5–7% [5, 24], whereas 11.6% of our cohort was IDH1-mutant. This discrepancy may reflect the inclusion of tumors arising from undiagnosed lower-grade gliomas, where IDH1 mutations occur in 73–88% of cases [7]. As primary and secondary HGGs are histologically indistinguishable without prior imaging, some tumors may have progressed from preexisting lower-grade lesions [25, 26]. To minimize bias from prior treatment, we included only new diagnoses and defined progression starting from initial surgery.

White patients were significantly more likely to have IDHwt glioblastomas, consistent with prior findings that gliomas are more common in non-Hispanic Whites [27, 28]. Research also suggests that non-Hispanic Whites experience worse outcomes despite standardized treatment [28–30]. Meanwhile, Hispanic and Mexican Hispanic patients may have higher rates of IDH1/IDH2 mutations [31, 32]. Thus, the observed racial distribution of IDH1 mutations in our cohort aligns with prior epidemiologic patterns.

Unexpectedly, Black race, rather than White race, predicted a shorter time to first recurrence. Multivariate analysis included only variables differing significantly between molecular groups, limiting confounding. While IDH1wt status was associated with recurrence at 6.1 months (p = 0.0491), Black race was similarly associated with a 7.3-month interval (p = 0.0238). As a multi-institutional study, these disparities are unlikely to stem from a single care team. Given the small sample size, these findings should be viewed as exploratory. Larger studies are needed to clarify whether this reflects biologic differences, access barriers, or other sociodemographic factors.

Although prior studies have examined IDH status and recurrence, most were limited to first recurrence or predated the 2021 WHO CNS5 classification [32]. Our inclusion of second recurrence data adds to the limited molecularly stratified literature. All institutions used the RANO 2.0 criteria to define recurrence, enhancing consistency and comparability across sites [20].

Due to strict inclusion criteria, our analytic cohort comprised less than 40% of the initial cohort. While this reduced statistical power, it was necessary for reliable recurrence analysis. Given the lack of consensus on managing recurrent HGG [33–36], this study provides valuable insight into recurrence patterns. Recent studies support reoperation at first recurrence [14, 37], and in our cohort, over half of recurrences were managed surgically. However, there were no statistically significant differences in surgical management between IDH1-mutant astrocytoma and IDHwt GBM. To avoid overfitting, only variables significantly different between groups were included in the regression model. Karschnia et al. [37] from the RANO resect group recently reinforced the prognostic relevance of resection extent at recurrence, proposing standardized classifications to guide care. Future studies with larger cohorts are needed to determine its independent effect. Given that only 43 patients experienced a second recurrence—including just 8 with IDH-mutant astrocytomas—all multivariable analyses for this outcome should be considered exploratory. These findings offer early insight into recurrence timing beyond first progression but require validation in prospective studies.

This study has several limitations. Its retrospective design introduces risks of selection bias, confounding, and information bias. Although all patients were treated according to NCCN-concordant protocols, recurrence management was not fully standardized. We accounted for this by incorporating recurrence treatments into exploratory multivariate models. The inclusion criteria may have selected for patients with higher functional status and better access to care, while excluding those lost to follow-up or who died before recurrence may have skewed the cohort toward more indolent disease. Chemotherapy and radiation were treated as variables, which do not capture differences in timing, dosing, or agent selection—particularly relevant after multiple recurrences, when care becomes increasingly individualized. In retrospective, multi-institutional datasets, it is difficult to fully account for such variability. While biopsy rates did not differ significantly between molecular groups, they were numerically higher in the IDH-mutant astrocytoma cohort. Given the association between biopsy and shorter progression-free survival [38], this variability may have introduced additional confounding and warrants further exploration in future stratified analyses.

Survival analyses were not conducted using Kaplan–Meier or log-rank methods, as only patients with confirmed recurrences were included, resulting in an uncensored dataset. Recurrence intervals were instead analyzed directly using Cox regression, reflecting available data and preserving statistical integrity.

An additional limitation was the inability to stratify IDH-mutant astrocytomas by WHO grade. While grade distinctions have prognostic value, grades 3 and 4 are often treated similarly in practice. This study sought to characterize recurrence patterns in a real-world clinical context rather than define grade-specific biology. Despite limitations, the findings offer meaningful insights into recurrence dynamics in actively managed HGG patients.

We did not analyze overall survival due to variability in how institutions recorded the date of death. Similarly, post-progression survival was not evaluated. In some cases, death dates came from family reports or death certificates, introducing further inconsistencies. Including these outcomes could have skewed survival estimates. Instead, we focused on recurrence intervals—a clinically relevant metric, as HGG recurrence is typically symptomatic [39]. Understanding the timing of recurrence, rather than just terminal outcomes, is essential for treatment planning and improving quality of life.

Given the challenges of tracking second recurrences, few studies have examined this disease stage. Our cohort of 43 patients with confirmed second recurrences offers a unique perspective. Among these, patients with IDH1-mutant astrocytomas had a PFS of 17.5 months initially, which declined to 10.8 months after first recurrence. By comparison, IDHwt GBM patients had a first recurrence interval of 9.8 months and a second of 8.1 months. The greater decline among IDH-mutant astrocytomas (6.7 months vs. 1.7 months) suggests that the early protective benefit of IDH1 mutations may diminish over time. One explanation is the accumulation of additional mutations that accelerate tumor progression [40, 41]. This aligns with prior work by Miller et al., which showed accelerated progression in IDH-mutant astrocytomas after first recurrence, marked by shortened second progression-free intervals [15].

MGMT methylation is another key factor influencing tumor behavior. Epigenetic silencing of MGMT is associated with prolonged survival in temozolomide-treated patients [41]. While MGMT methylation significantly prolonged time to first recurrence, it did not affect time to second recurrence in our study. This may reflect limited sample size or progressive genetic evolution. As MGMT status was unavailable in 38% of HGG patients and 50% of IDH1-mutant astrocytomas, it was excluded from the primary model. Nonetheless, our findings suggest that while MGMT methylation confers initial benefit, its effect may wane over time. Whether salvage therapies should be stratified by MGMT status at recurrence remains an open question for future trials.

## Conclusion

IDH mutation status predicted a longer time to first recurrence but did not impact time to second recurrence. Wild-type IDH and Black race were associated with shorter time to first recurrence. These findings, while limited by retrospective design, small sample size, and limited subgroup power, underscore the evolving nature of HGG recurrence. They also highlight the need to explore genetic and socioeconomic influences on disease progression. Larger, prospective studies with molecularly stratified cohorts are needed to validate these results and inform individualized treatment and surveillance across the disease course.

## Data Availability

The datasets generated and analyzed during this study are not publicly available but are available from the corresponding author upon reasonable request.
